# Supplementary figures and images for: Class incremental learning of remote sensing images based on class similarity distillation
Source: PeerJ Comput Sci. 2023 Sep 27;9:e1583. doi: 10.7717/peerj-cs.1583 (PMC10557500; doi:10.7717/peerj-cs.1583)

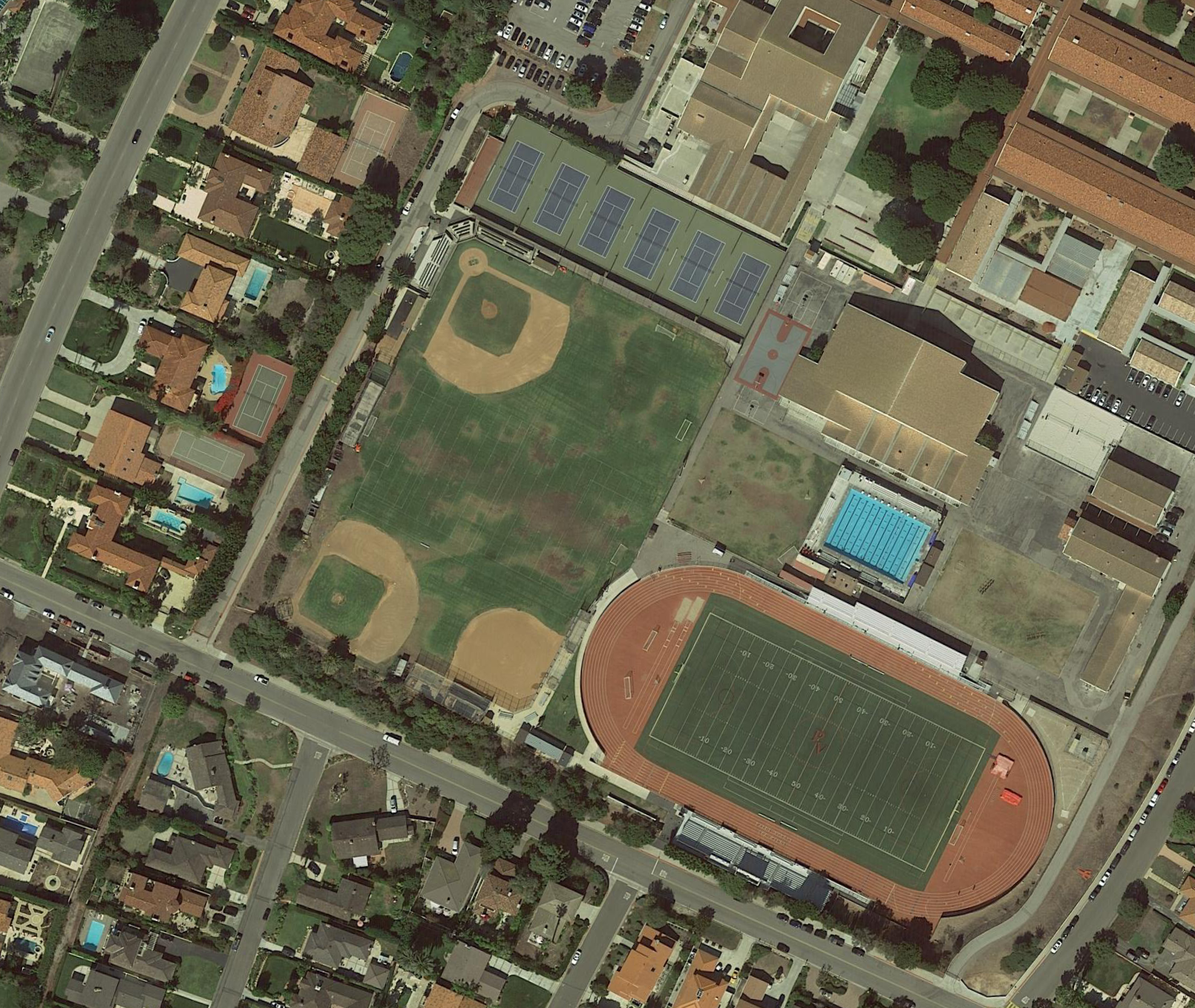

Supplement: Supplemental Information 1 [file peerj-cs-09-1583-s001.zip › code/attention/4.png]

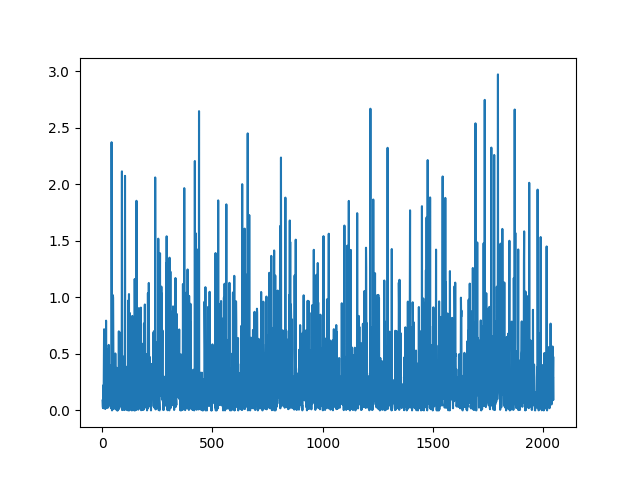

Supplement: Supplemental Information 1 [file peerj-cs-09-1583-s001.zip › code/attention/features_whitegirl/f9_avgpool.png]

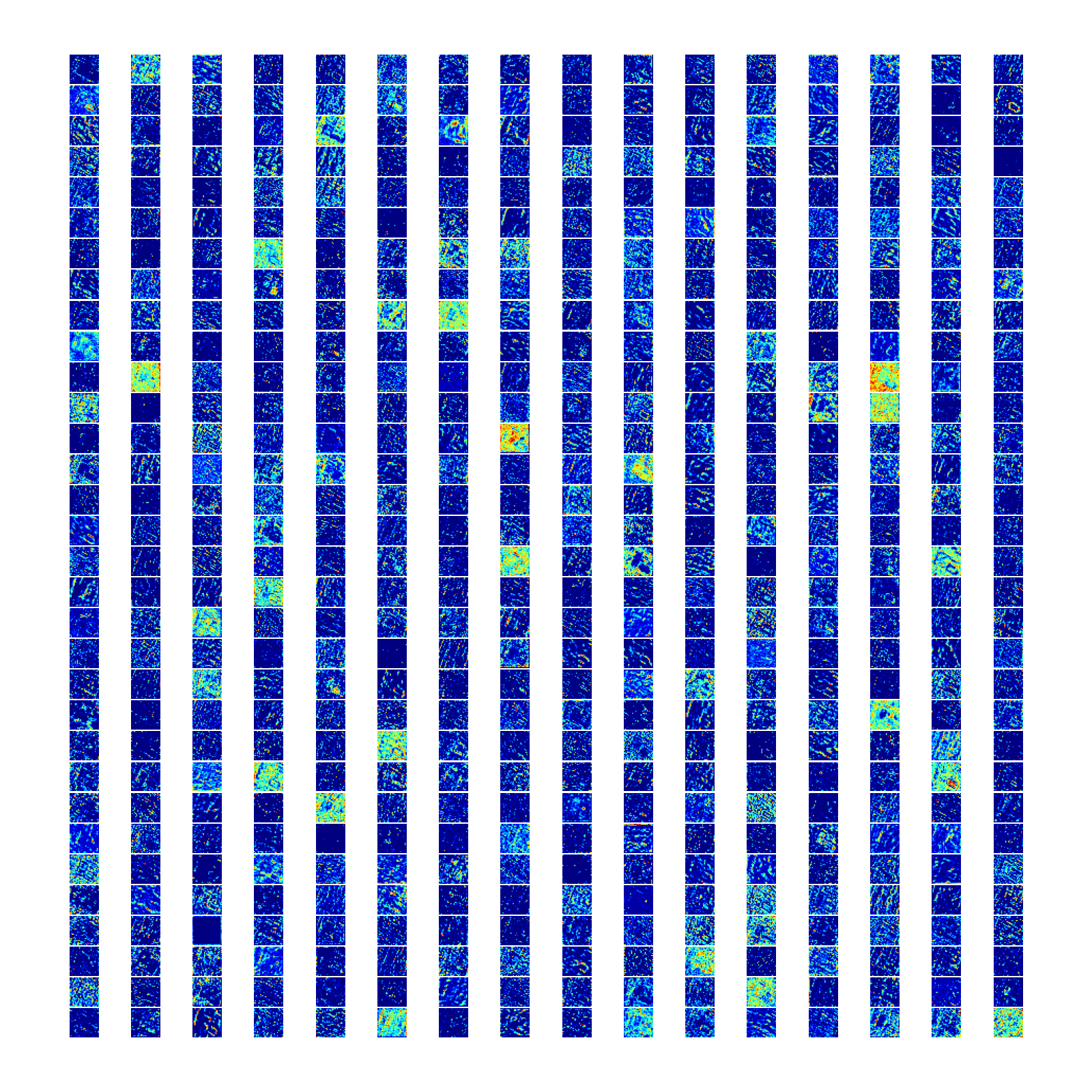

Supplement: Supplemental Information 1 [file peerj-cs-09-1583-s001.zip › code/attention/features_whitegirl/f6_layer2.png]

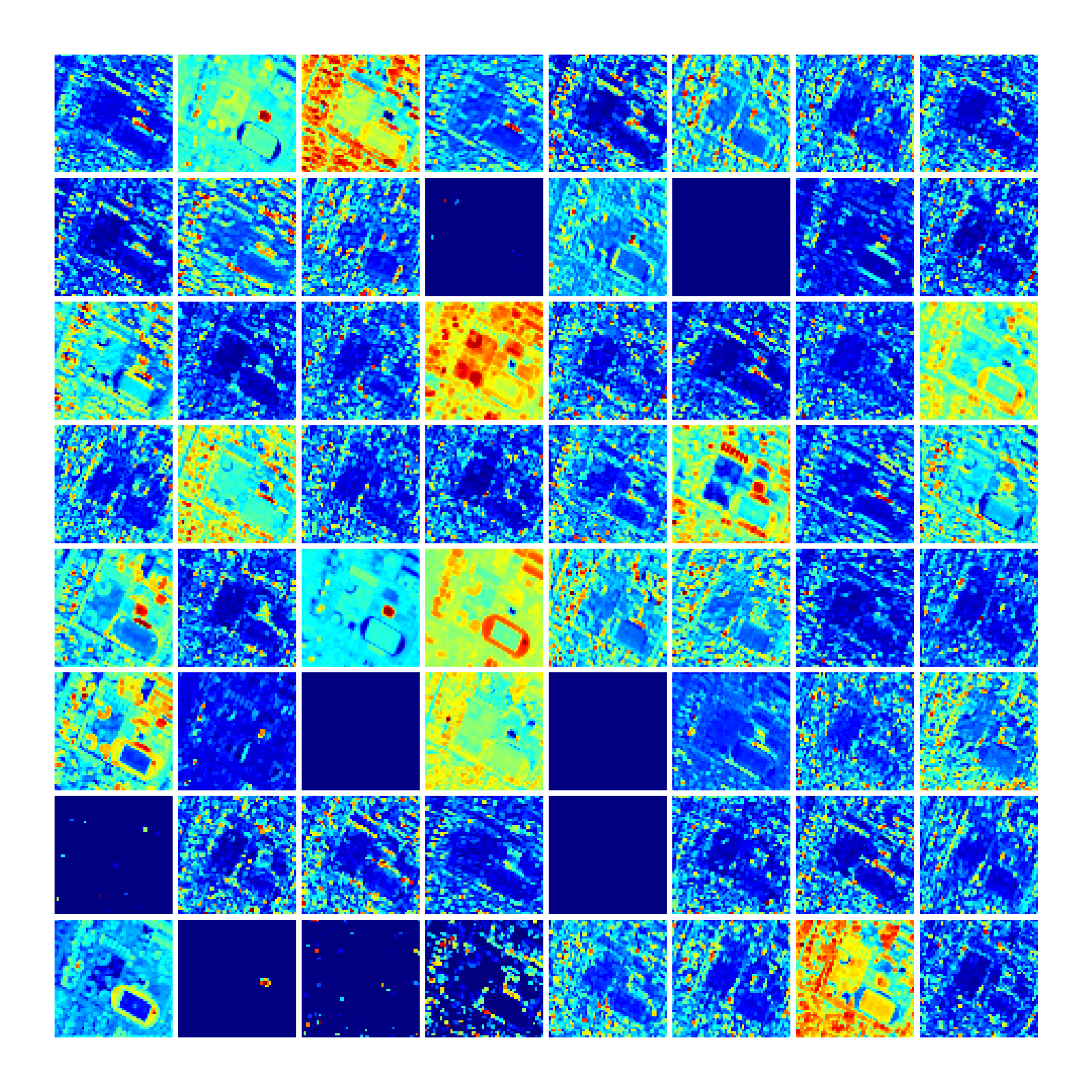

Supplement: Supplemental Information 1 [file peerj-cs-09-1583-s001.zip › code/attention/features_whitegirl/f4_maxpool.png]

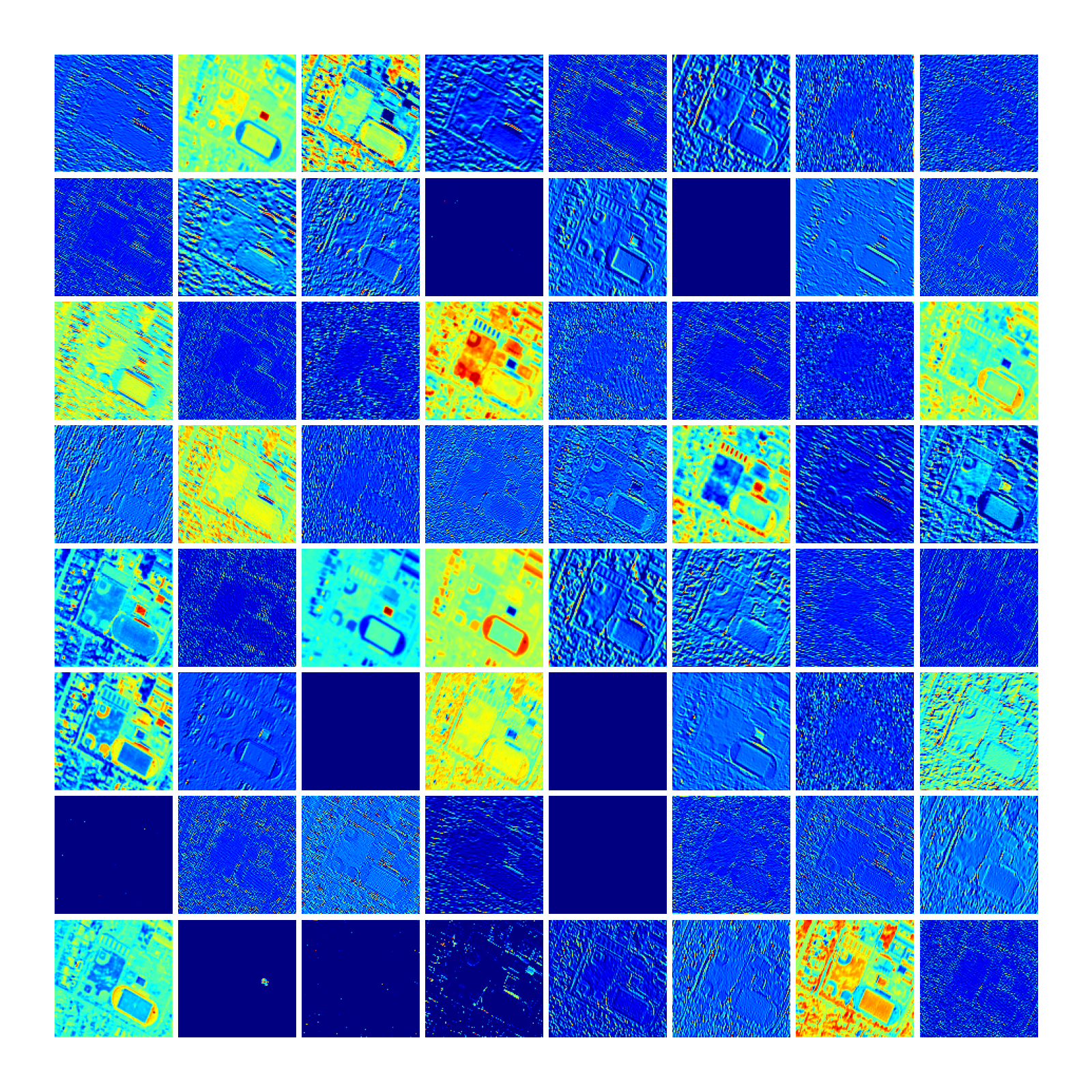

Supplement: Supplemental Information 1 [file peerj-cs-09-1583-s001.zip › code/attention/features_whitegirl/f3_relu.png]

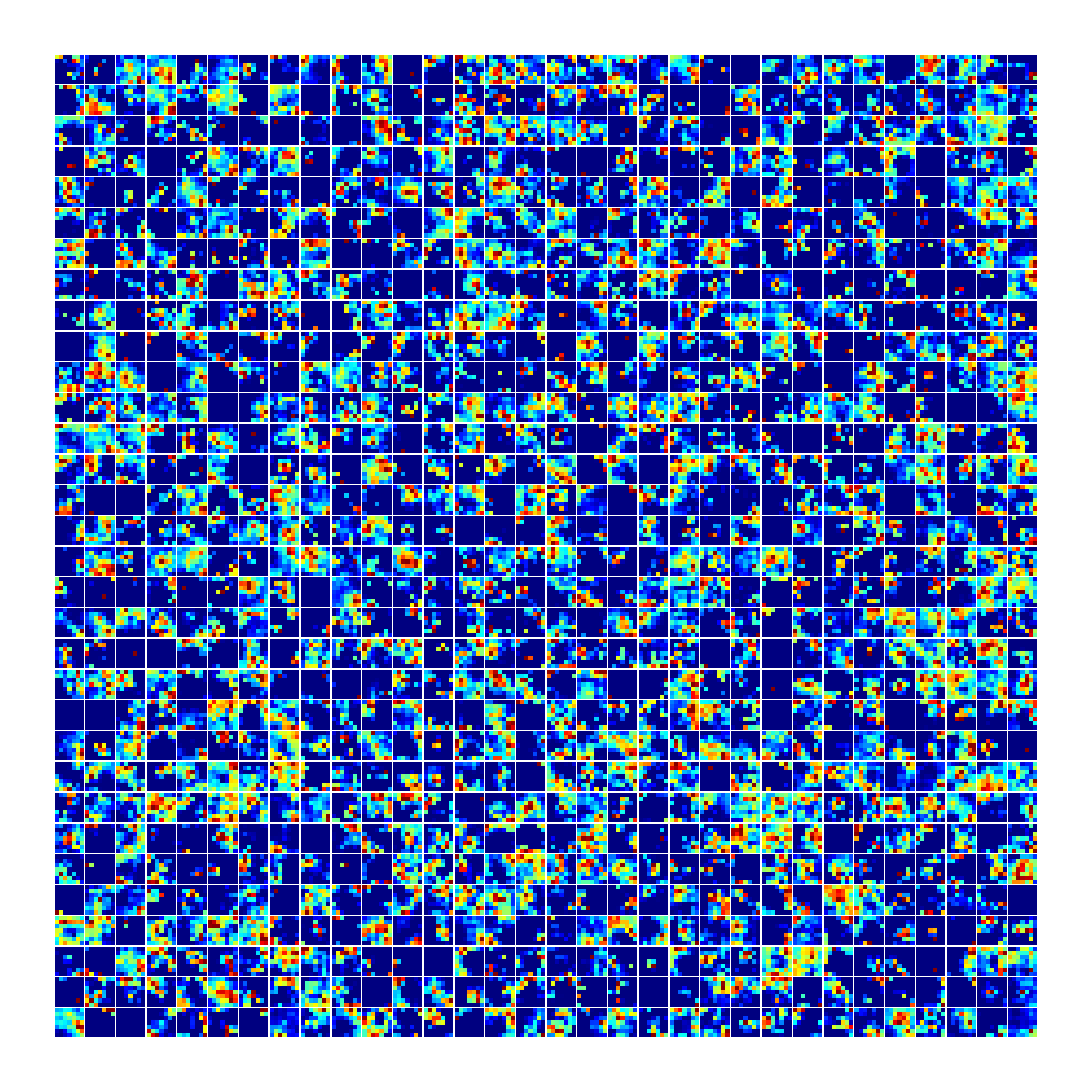

Supplement: Supplemental Information 1 [file peerj-cs-09-1583-s001.zip › code/attention/features_whitegirl/f8_layer4_2.png]

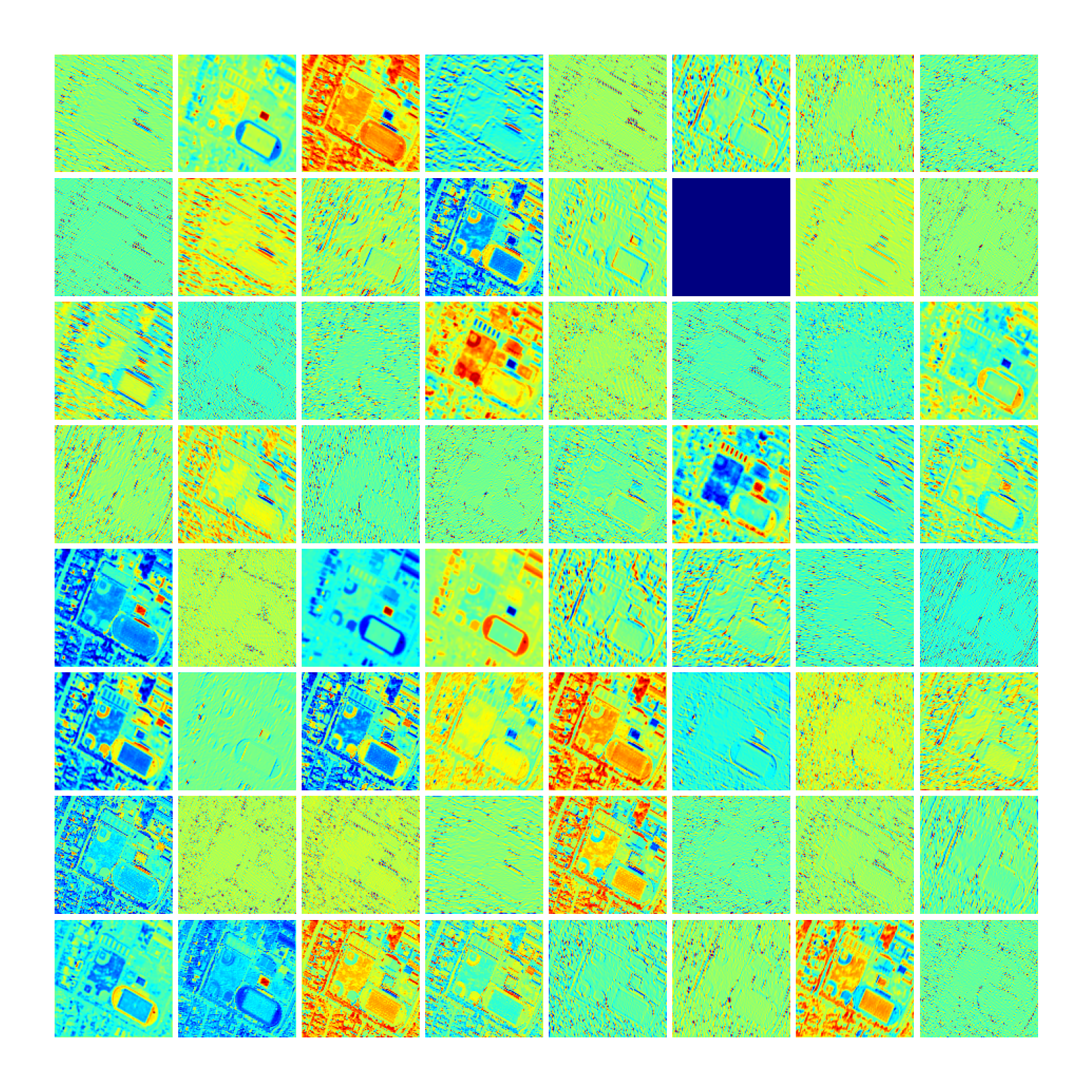

Supplement: Supplemental Information 1 [file peerj-cs-09-1583-s001.zip › code/attention/features_whitegirl/f2_bn1.png]

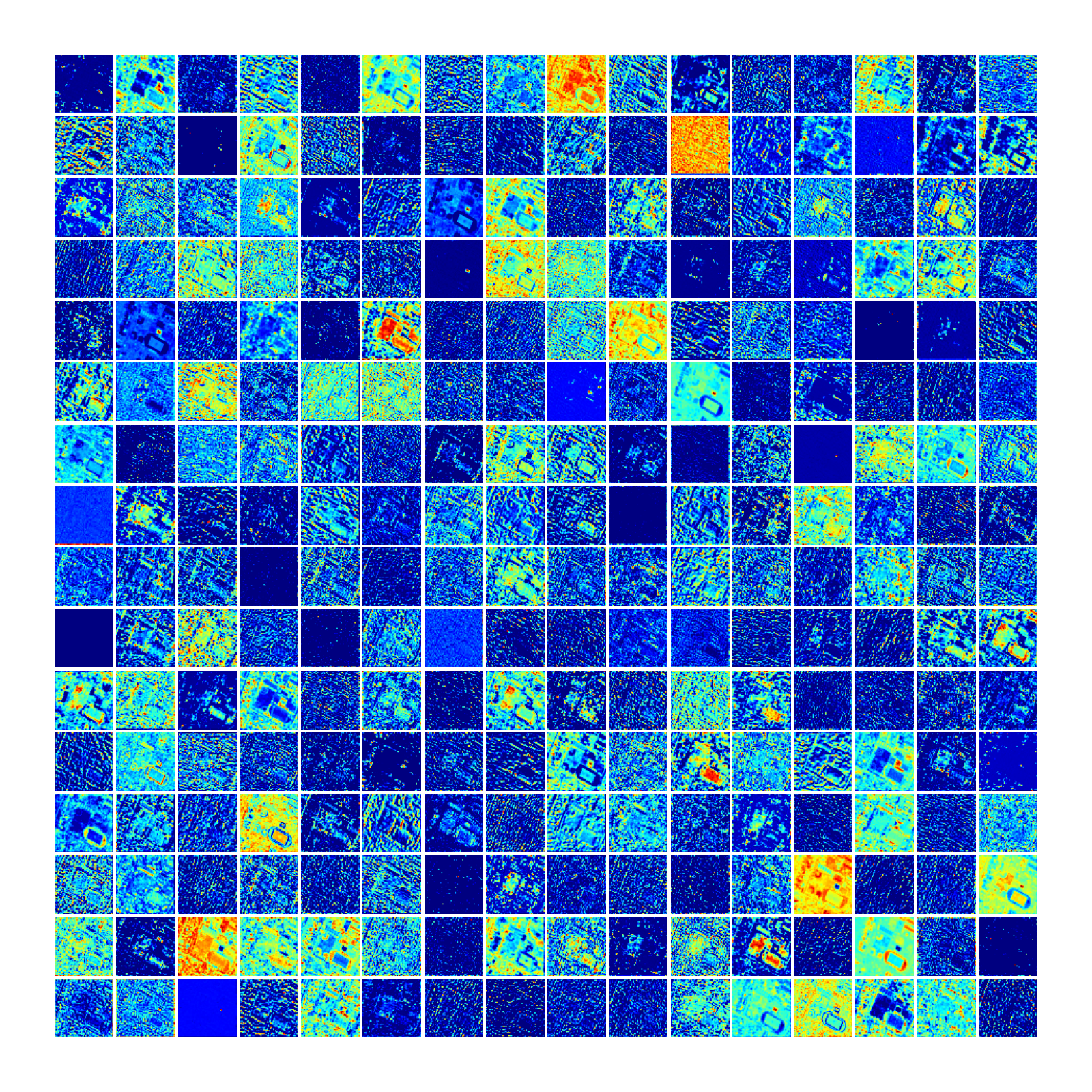

Supplement: Supplemental Information 1 [file peerj-cs-09-1583-s001.zip › code/attention/features_whitegirl/f5_layer1.png]

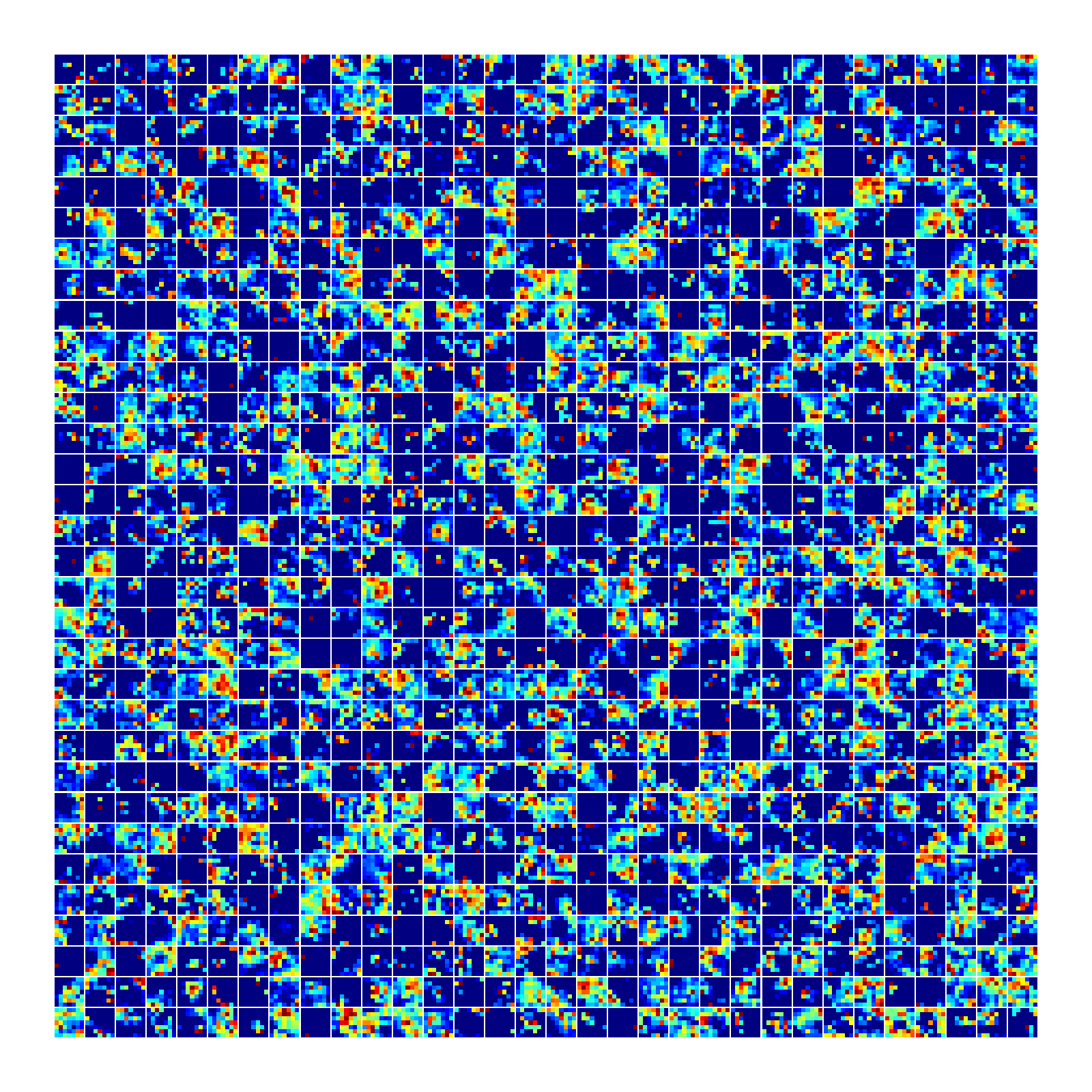

Supplement: Supplemental Information 1 [file peerj-cs-09-1583-s001.zip › code/attention/features_whitegirl/f8_layer4_1.png]

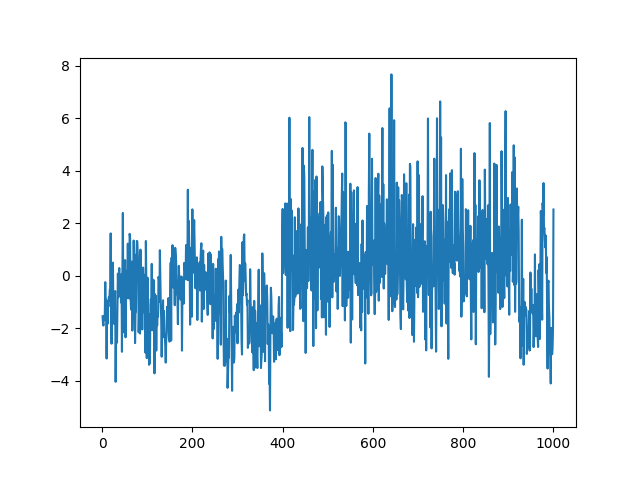

Supplement: Supplemental Information 1 [file peerj-cs-09-1583-s001.zip › code/attention/features_whitegirl/f10_fc.png]

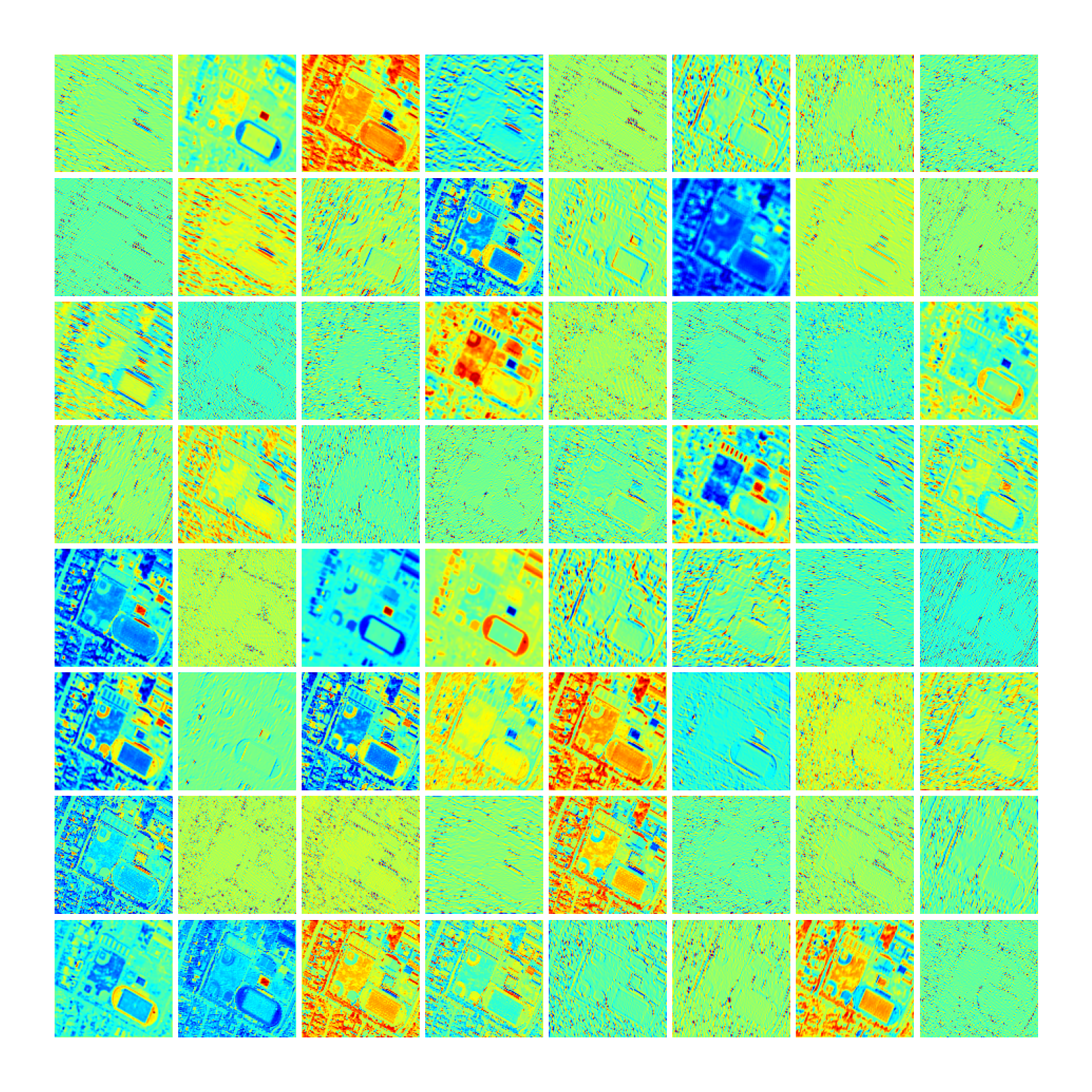

Supplement: Supplemental Information 1 [file peerj-cs-09-1583-s001.zip › code/attention/features_whitegirl/f1_conv1.png]

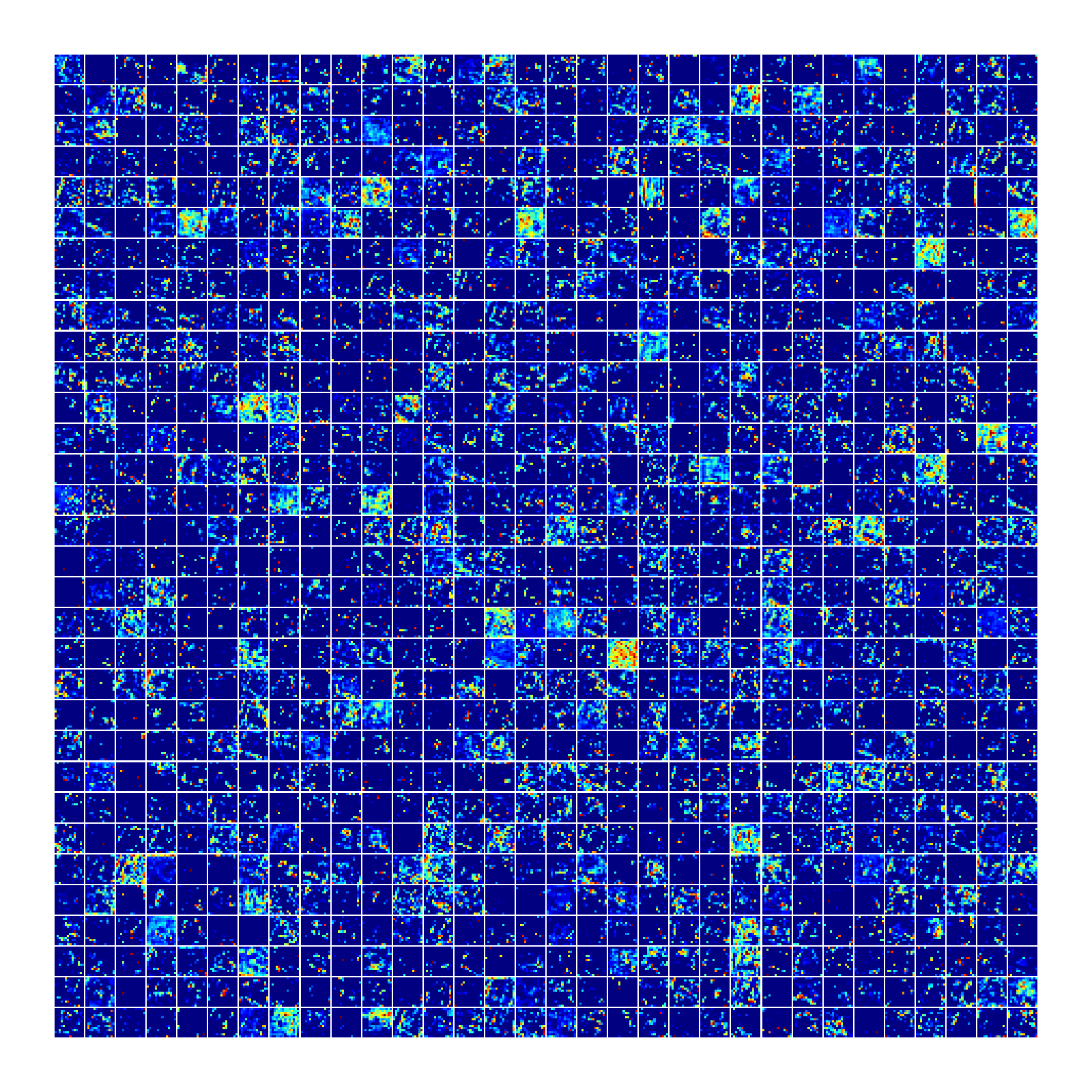

Supplement: Supplemental Information 1 [file peerj-cs-09-1583-s001.zip › code/attention/features_whitegirl/f7_layer3.png]
